# THE RELATIONSHIP BETWEEN HURRICANE STRESSORS AND COGNITION IN PUERTO RICAN OLDER ADULTS

**DOI:** 10.1093/geroni/igad104.0847

**Published:** 2023-12-21

**Authors:** Sheila Thompson, Victoria Guinn, Erin Ballard, Michael Crowe, Lisa Brown

**Affiliations:** Palo Alto University, Palo Alto, California, United States; Palo Alto University, Palo Alto, California, United States; University of Alabama at Birmingham, Birmingham, Alabama, United States; University of Alabama-Birmingham, Birmingham, Alabama, United States; Palo Alto University, Palo Alto, California, United States

## Abstract

Acute and chronic stress exposure negatively impact cognition in older adults. There is little research on this relationship within the context of an acute disaster. This presentation explores the relationship between perceived stress, hurricane stressors, subjective cognition, and cognition in Puerto Rican older adults post-Hurricane Maria. 613 Puerto Rican older adults completed Wave 3 measures for the longitudinal PREHCO study. Perceived stress was measured via a 4-item version of the Perceived Stress Scale. Hurricane stressors were classified as three factors: Home Damage, Family/Friends Network, and Work/Health. Subjective cognition was measured by asking the participant to rate their memory based on a 5-point Likert scale. Cognition was measured via the Telephone Interview for Cognitive Status assessment and the Minimental Cabán. Analyses included two multiple linear regressions with cognitive status and cognitive functioning as the outcomes, respectively, while controlling for age, depressive symptoms, education, gender, and race. There was a significant relationship between higher concern for others post-hurricane and worse generalized cognitive functioning (p<0.05, t=-2.04). Higher levels of cognitive status were significantly associated with better perceptions of memory function (p= 0.015, t=-2.43). Yet higher levels of perceived stress were significantly associated with worse perceptions of memory function (p<0.001, t=7.037). Older adult Puerto Ricans presented with worse cognitive functioning when they were worried about their loved ones’ well-being. Even those who maintained an intact cognitive status believed their memory was worse when they were experiencing more stress. This highlights the importance of differentiating the impact of stress on cognitive ability from subjective cognition.

